# Enhanced pentose phosphate pathway activity promotes pancreatic ductal adenocarcinoma progression via activating YAP/MMP1 axis under chronic acidosis

**DOI:** 10.7150/ijbs.69526

**Published:** 2022-03-06

**Authors:** Siyuan Chen, Bo Ning, Jinwen Song, Zihan Yang, Li Zhou, Zhiji Chen, Linhong Mao, Hongtao Liu, Qingliang Wang, Song He, Zhihang Zhou

**Affiliations:** 1 Department of Gastroenterology, the Second Affiliated Hospital of Chongqing Medical University, China.; 2 Department of Infectious Diseases, the Fifth Medical Center of Chinese PLA General Hospital, National Clinical Research Center for Infectious Diseases, Beijing, China.; 3 Department of Biomedical Science, City University of Hong Kong, Hong Kong SAR, China.; 4 Department of Pathology, the Second Affiliated Hospital of Chongqing Medical University, China.

**Keywords:** Acidic microenvironment, PDAC, Metastasis, MMP1, Hippo signaling, AMPK

## Abstract

**Background:** Acidic microenvironment is a common physiological phenomenon in tumors, and is closely related to cancer development, but the effects of acidosis on pancreatic adenocarcinoma (PDAC) remains to be elucidated.

**Methods**: Metabonomic assay and transcriptomic microarray were used to detect the changes of metabolites and gene expression profile respectively in acidosis-adapted PDAC cells. Wound healing, transwell and *in vivo* assay were applied to evaluate cell migration and invasion capacity. CCK8 and colony formation assays were performed to determine cell proliferation.

**Results:** The acidosis-adapted PDAC cells had stronger metastasis and proliferation ability compared with the control cells. Metabonomic analysis showed that acidosis-adapted PDAC cells had both increased glucose and decreased glycolysis, implying a shift to pentose phosphate pathway. The metabolic shift further led to the inactivation of AMPK by elevating ATP. Transcriptomic analysis revealed that the differentially expressed genes in acidosis-adapted cells were enriched in extracellular matrix modification and Hippo signaling. Besides, MMP1 was the most upregulated gene in acidosis-adapted cells, mediated by the YAP/TAZ pathway, but could be reduced by AMPK activator.

**Conclusion**: The present study showed that metabolic reprogramming promotes proliferation and metastasis of acidosis-adapted PDAC cells by inhibiting AMPK/Hippo signaling, thus upregulating MMP1.

## Introduction

To adapt to the adverse living environment caused by unlimited proliferation, the metabolic mode of tumor cells presents a background dependent flexibility 1. Tumor cells turn to aerobic glycolysis to accommodate rapid proliferation, which generates a large amount of lactic acid. In addition, proton and carbon dioxide are respectively produced in the process of ATP consumption and oxidative phosphorylation. All of these acidic materials are expelled into the extracellular microenvironment and retained in this space due to hypoperfusion, leading to a decrease in pH value of the tumor microenvironment to 6.4-7, namely, acidosis 2. Acidosis is a selective pressure for tumor cells, and those that survive have additional advantages, such as enhanced proliferation, metastasis, and drug resistance 3. This explains why hepatocellular carcinoma cells proliferate and metastasize under an acidosis condition 4. Furthermore, Faes S et al. revealed that the acidic tumor microenvironment made mTORC1 inhibitors ineffective 5. Moreover, lower pH value promotes malignant glioma 6, breast cancer 7, colon cancer 8, and lung cancer 9 progression. Our previous work found that the acid sensing ion channel, ASIC2, promotes colorectal cancer cell invasion under acidosis 10, and acid-adapted colon cancer cells have stronger metastasis capacity 11. As a widely recognized pro-tumor effect, acidosis has been applied as a marker for prognosis determination 12 and treatment target 13. However, the underlying mechanism of acidosis should be further demonstrated, especially for chronic acidosis.

The acidic microenvironment can also in turn induce a metabolic shift. Minnan Zhao and colleagues found that acidosis-adapted colorectal cancer cells (HCT15, HCT116 and LoVo) maintain a higher level of reduced glutathione to reduce the acid-induced reactive oxygen species via the upregulation of CD44 and glutathione reductase (GSR) 14. Additionally, the acidosis-adapted colorectal cancer cell (HCT116), cervical cancer cell (SiHa) and pharynx squamous cell carcinoma cell (FaDu) all exhibited a turnover from glycolysis to glutamine metabolism, which was mediated by hypoxia inducible factor-2α (HIF2α) 15. The same research group further revealed that the acidic microenvironment profoundly reprograms the metabolism of these cancer cells toward fatty acid oxidation by inducing histone deacetylation and downregulating acetyl-CoA carboxylase, or ACC2 16, 17. As metabonomic technology developed, it came to reveal significant metabolic changes under acute or chronic acidosis. Recently, it was reported that acute acidic treatment (pH 6.8 for 36 hours) increased the *de novo* purine nucleotide biosynthesis activity in glioma stem cells by upregulating glucose-6-phosphate dehydrogenase (G6PD) or G6PD expression 18. AMP-activated protein kinase (AMPK) is an energy sensor that monitors the ratio of AMP:ADP:ATP in eukaryotic cells. AMPK has generally been considered a tumor suppressor, as it is a putative substrate of tumor suppressor, Liver kinase B1 (LKB1) 19. AMPK phosphorylates angiomotin like 1 (AMOTL1), an adaptor protein in the Hippo-Yap pathway, and thus blocks Yes1 associated transcriptional regulator (YAP) activity, dampening cancer cells proliferation and survival 20. Further work should be done to elucidate the mechanism of metabolic reprogramming under chronic acidosis and its correlation with tumor progression, especially in the context of pancreatic ductal adenocarcinoma (PDAC), one of the most lethal malignancies 21.

In this study, we generated acidosis-adapted PDAC cells by continuously culturing with an acidic medium (pH 6.5) for 3 months. We found that cell proliferation and metastasis ability increased, and matrix metalloproteinase-1 (MMP1) expression was profoundly upregulated. Furthermore, metabonomic analysis showed a shift towards pentose phosphate pathway (PPP) metabolism as intracellular glucose was increased, while glycolysis was significantly inhibited in acidosis-adapted PDAC cells. This shift further led to the inhibition of AMPK signaling by increasing ATP level. Inhibition of AMPK then increased the YAP and MMP1 expression, promoting invasion and metastasis. Our study demonstrated increased PPP metabolism promotes development of acidosis-adapted PDAC cells via AMPK/YAP/MMP1 axis, enriching the mechanism by which acidic environment promotes tumor progression.

## Materials and Methods

### Cell lines and cell culture

Human pancreatic cancer cell lines PANC-1 and SW1990 were obtained from the American Type Culture Collection (Manassas, VA, USA), where all of the cells were identified by short-tandem-repeat profiling and without mycoplasma contamination. Cells were cultured in Dulbecco's Modified Eagle Medium (DMEM) pH 7.4 or pH 6.5 (HyClone, Logan, UT, USA). All culture media were supplemented with 10% fetal bovine serum (Gibco, Rockville, MD, USA), 100 units/mL penicillin, and 100 μg/mL streptomycin (HyClone). All cells were grown in a 37 °C humidified atmosphere containing 5% CO2.

### Metabonomic analysis

The metabonomic analysis was done by Oebiotech (Shanghai, China). Briefly, 5×10^7^ cells were processed using ultrasonic homogenizer in a mixture of methanol and water (1/4, vol/vol). L-2-chlorophenylalanine (0.3 mg/mL) dissolved in methanol was used as the internal standard. The dried supernatant was then dissolved in pyridine containing 15 mg/mL methoxyamine hydrochloride. The samples were then subjected to LC-MS and GC-MS analysis. The obtained GC/MS raw data in .D format were transferred to .abf format via software Analysis Base File Converter for quick data retrieval. Then, data were imported into software MS-DIAL, which performs peak detection, peak identification, MS2Dec deconvolution, characterization, peak alignment, wave filtering, and missing value interpolation. Metabolite characterization was based on LUG database. The original LC-MS data were processed by the Progenesis QI V2.3 software (Nonlinear, Dynamics, Newcastle, UK) for baseline filtering, peak identification, integral, retention time correction, peak alignment, and normalization. Main parameters of 5 ppm precursor tolerance, 10 ppm product tolerance, and 5% product ion threshold were applied. Compound identification were based on precise mass-to-charge ratio (M/z), secondary fragments, and isotopic distribution using The Human Metabolome Database (HMDB), Lipidmaps (V2.3), Metlin, EMDB, PMDB, and self-built databases to do qualitative analysis. Variable Importance of Projection (VIP) values obtained from the OPLS-DA model were used to rank the overall contribution of each variable to group discrimination. A two-tailed Student's t-test was further used to verify whether the metabolites of difference between groups were significant. Differential metabolites were selected with VIP values greater than 1.0 and p-values less than 0.05.

### ATP detection assay

Luminescent ATP detection assay kit (Abcam, UK) was used to detect ATP level. In short, each group of cells were spread into 96 well plates with 1×10^5^ cells/well, and three multiple wells were set. After the cells adhered to the plate, removed the medium, added 100 μL fresh medium and 50 μL detergent into each well, 600r shaking for 5 min. Then added 50 μL substrate buffer into each well, 600r shaking for 5 min. Finally, luminescence was measured, and ATP content was calculated according to standard curve.

### Reactive Oxygen Species (ROS) assay

ROS assay kit was bought from Beyotime (Shanghai, China). The operation steps were carried out according to the S0033 manual. Briefly, after the cells adhered to the slides, the ROS probe was diluted with serum-free medium according to 1:1000 and incubated with cells at 37 °C for 20 minutes. After washing the cells with PBS for three times, ROS was detected by confocal fluorescence microscope at 522 nm.

### NADP+/NADPH assay

NADP+/NADPH detection was carried out according to the method of Beyotime S0179 (Shanghai, China). 200 μL of NADP+/NADPH Extract Buffer was added into 1×10^6^ cells. Cells were centrifuged at 12000g, 4 °C for 10 minutes after fully lysed on ice. 50 μL supernatant was transferred to 96 well plate. Another 100 μL supernatant was heated at 60 °C for 30 minutes to decompose NADP+, and then took 50 μL heated supernatant into 96 well plate. Incubating the plate at 37 °C in the dark for 10 minutes. 10 μL Determination Buffer was added to each well, incubated in the dark at 37 °C for 10-20 minutes. Finally, the absorbance was measured at 450 nm. NADP+/NADPH ratio was calculated by standard curve.

### Glucose-6-phosphate dehydrogenase (G6PDH) activity assay

Beyotime S0189 (Shanghai, China) was used to measure G6PDH activity. In short, 200 μL of G6PDH Extract Buffer was added to 1×10^6^ cells, after the cells were fully lysed on ice, 12000g centrifuged at 4 °C for 5-10 minutes. 50 μL supernatant was transferred into 96 well plate, and added with 50 μL G6PDH Detection Solution. The Mix was incubated at 37 °C in the dark for 10 minutes, and measured the absorbance at 450 nm. G6PDH activity was calculated by standard curve.

### Clinical PDAC samples

PDAC specimens were collected from 61 patients with PDAC from 2011 to 2015 at the Second Affiliated Hospital of Chongqing Medical University after informed consent was obtained from all patients. The patients did not receive chemotherapy or radiotherapy before surgery. The diagnoses of PDAC were made independently by at least two histopathologists. This study was carried out according to the principles of the Helsinki Declaration and approved by the Ethical Committee of the Second Affiliated Hospital of Chongqing Medical University ((2019)133).

### Lentivirus infection

Lentiviruses carrying small hairpin RNA (shRNA) sequence of human MMP1 were purchased from Obio Company (Shanghai, China). Sequences for MMP1 shRNA and control were as follows: shRNA-1 (5'-GCCTTCCAACTCTGGAGTAAT-3'), shRNA-3 (5'-GCGTGTGACAGTAAGCTAACC-3'), and control (5'-AAACGTGACACGTTCGGAGAA-3'). Cells were cultured in 24-well plates at 1×10^5^ cells/well, and lentivirus was added into the medium separately (MOI=20). The medium was refreshed after 12h. Puromycin was used to screen the stable cells after 72 hours of infection.

### mRNA expression microarray

RNA from acidosis-adapted PANC-1 cells and parental cells was extracted as mentioned in supplementary materials and methods. Then the RNA was incubated with Agilent mRNA+LncRNA microarray (Capitalbiotech, Beijing, China). The differentially expressed genes were screened by the following criterion: fold change larger than 2 and p value less than 0.05. Then these genes were applied to enrichment analysis by KEGG and GO database.

### Tail vein injection mouse model

Briefly, 1×10^7^ tumor cells (SW1990-NA-NC, SW1990-NA-shMMP1, SW1990-AA-NC, SW1990-AA-shMMP1) were suspended in 100μL PBS and injected into nude mice from the tail vein. The mice were sacrificed 4 weeks after injection and lungs were collected for pathological test. The lungs were dissected into 5 mm thick pieces and each piece was subjected to dehydration, embedding. Pathological section with 4 μm thickness were made and stained with H&E. The metastatic foci were identified by a pathologist.

Other methods and materials were in the supplementary data.

### Statistical analysis

All values are presented as means ± standard deviation 22. All data were obtained from at least three repetitions of each experiment. Prism 8.0 (GraphPad, USA) software was used to analyze the data. All data are shown as mean ± SD. Student's t-test was used to analyze differences between two groups. One-way ANOVA was used to compare three or more groups. Probability values less than 0.05 was considered statistically significant.

## Results

### Metabolic shift towards pentose phosphate pathway in acidosis-adapted PDAC cells inhibited AMPK signaling

**Figure 1:**
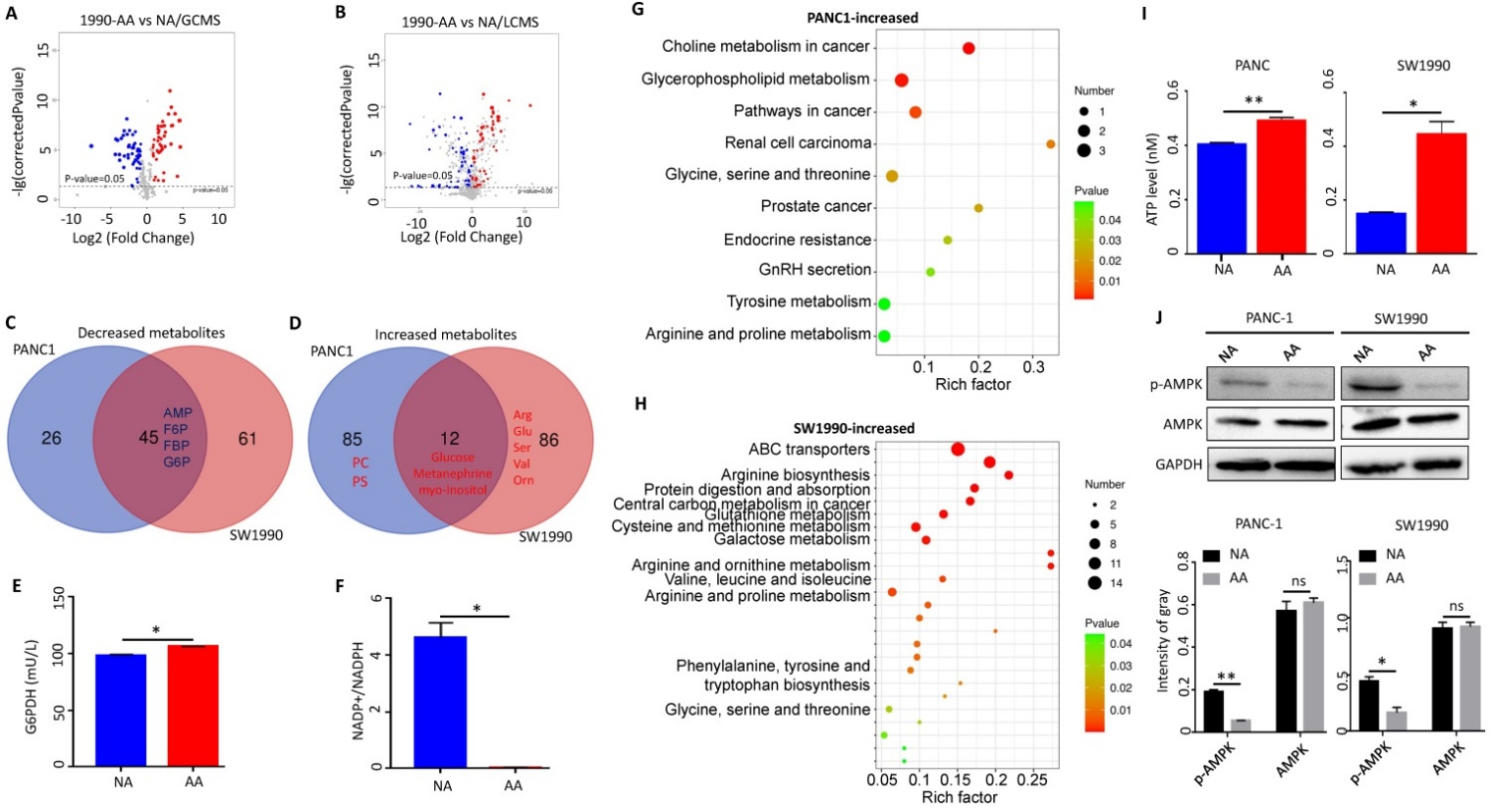
** Metabolic shift towards pentose phosphate pathway in acidosis-adapted PDAC cells inhibited AMPK signaling. (A)** The GC-MS detected changes of metabolites in SW1990-NA and SW1990-AA cells. **(B)** The LC-MS revealed changes of metabolites in SW1990-NA and SW1990-AA cells. **(C & D)** Analysis of decreased and increased metabolites in PANC-1-AA vs. SW1990-AA cells. **(E)** Detection of G6PDH activity in SW1990-NA and SW1990-AA cells. **(F)** Detection of NADP+/NADPH ratio in SW1990-NA and SW1990-AA cells. **(G & H)** Pathway changed in PANC-1-AA and SW1990-AA cells. **(I)** ATP increased in PANC-1-AA and SW1990-AA cells. **(J)** The activation of AMPK was detected by WB in acidosis-adapted PDAC cells and their control cells (the upper panel). Gray value analysis of WB is the lower panel. G6PDH, glucose-6-phosphate dehydrogenase; NADPH, reduced nicotinamide adenine dinucleotide phosphate; AMP, adenosine monophosphate; F6P, fructose-6-phosphate; F1P, fructose-1-phosphate; FBP, fructose-1,6-bisphosphate; G6P, glucose-6-phosphate; PC, phosphatidylcholine; PS, phosphosphingolipids. *, P<0.05; **,* P*<0.01; N=3.

Acidosis-adapted PDAC cells (PANC-1-AA and SW1990-AA) were obtained by culturing in acidic medium of pH 6.5 for three months, and the control cells were cultured in DMEM at pH 7.4 (PANC-1-NA and SW1990-NA). Non-targeting metabonomic analysis, including LC-MS and GC-MS, was applied to reveal the metabolic changes in acidosis-adapted PDAC cells (SW1990 and PANC1). The GC-MS revealed that 99 and 69 metabolites were respectively changed in SW1990 (Fig. 1A) and PANC1 cells (Supplementary Fig. 1A). Accordingly, LC-MS revealed that 105 and 99 metabolites were respectively changed in SW1990 (Fig. 1B) and PANC1 cells (Supplementary Fig. 1B). The common decreased metabolites, overlapped from LC-MS and GC-MS, include intermediates of glycolysis such as fructose-6-phosphate (F6P), fructose-1-phosphate (F1P), fructose-1,6-bisphosphate (FBP), glucose-6-phosphate (G6P), and adenosine monophosphate (AMP) (Fig. 1C). In contrast, a few metabolites were increased and included glucose, metanephrine and myo-inositol (Fig. 1D). The fact that acidosis-adapted PDAC cells showed both increased glucose and decreased glycolysis implies that these cells turn to the PPP, the opposite of glucose catabolism. The PPP process supplies NADPH and ribose5-phosphate (R5P) 23. These two metabolites are vital for nucleic acid synthesis, fatty acids, sterols, nucleotides and non-essential amino acids 24. We detected the key enzyme G6PDH, which in the first step of PPP pathway, and found that its activity increased significantly in acidosis-adapted cells (Fig. 1E) and the ratio of NADP+/NADPH, the downstream of G6PDH, was decreased significantly (NADPH increased in acidosis-adapted cells) (Fig. 1F). Moreover, compared with control cells, the level of ROS also reduced in acidosis-adapted cells (Supplementary Fig. 1C).

Besides, other downstream products of PPP were increased in acidosis-adapted PDAC cells, although with some difference between cells. The metabolites that were only increased in SW1990 cells include arginine, serine, glutamine, isoleucine, guanosine, ornithine, citrulline, glutathione, and valine (Fig. 1D). The metabolites that were only increased in PANC1 cells include phosphatidylcholine (PC), phosphosphingolipids (PS), glutaric acid, fumaric acid, tromethamine, testosterone, and methylamine (Fig. 1D). The increased metabolites in PANC1 cells were enriched during choline metabolism in cancer, glycerophospholipid metabolism, glycine, serine and threonine metabolism and arginine/proline metabolism (Fig. 1G). In contrast, the metabolites that were increased in SW1990 cells were mainly enriched in amino acid metabolism pathways such as arginine biosynthesis, glutathione metabolism, cysteine and methionine metabolism, and valine/leucine/isoleucine biosynthesis (Fig. 1H). As AMP was decreased in acidosis-adapted PDAC cells and because altered metabolites are related to energy production, we detected that the intracellular ATP level was higher in acidosis-adapted cells (Fig. 1I). These results revealed that the PDAC cells have different compensatory metabolic ways to increase intracellular ATP level after enhancing PPP. AMPK signaling is the sensor for ATP/AMP ratio. We found that the phosphorylation level of AMPK was lower in acidosis-adapted cells than parental cells (Fig. 1J). Altogether, these results demonstrated that the metabolic reprogramming towards PPP in acidosis-adapted PDAC cells inhibited AMPK signaling. Since glucose was increased in acidosis-adapted cells, we treated each PDAC cells with 10 mM glucose for 24 h, and found that AMPK activity was decreased**,** but there was no significant change in ATP (Supplementary Fig. 1D & E). However, glucose has a wide range of effects on metabolism, which may vary with time and dose, therefore the role of glucose in adapting to acidic microenvironment needs to be further explored.

### Acidosis-adapted PDAC cells exhibited enhanced migration and invasion ability

**Figure 2:**
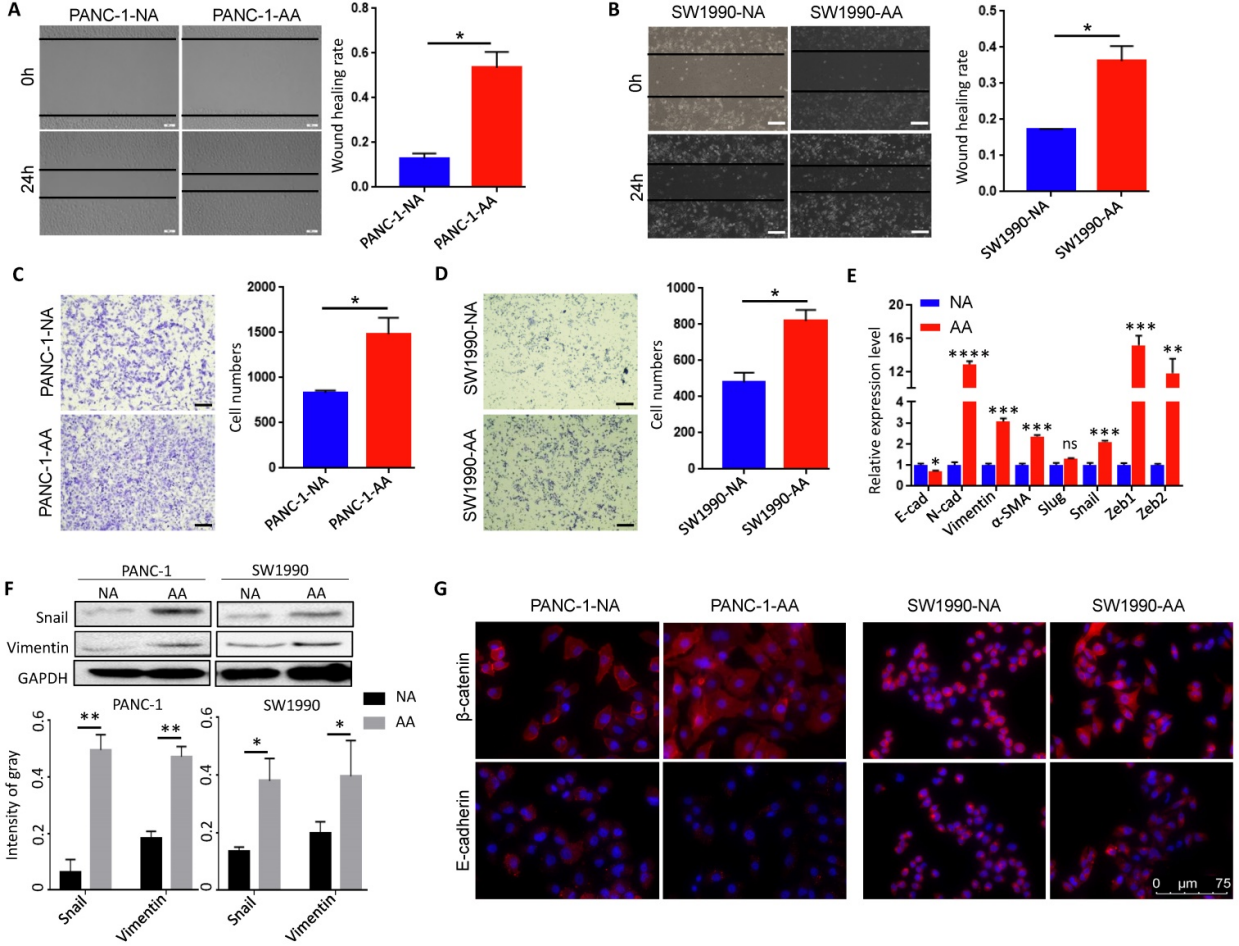
** Migration and invasion capacity was enhanced in acidosis-adapted PDAC cell. (A & B)** Wound healing assays reflected migration in PANC-1-AA, SW1990-AA and NA cells. **(C & D)** Transwell assay showed invasion abilities in PANC-1-AA, SW1990-AA and NA cells. The scale bars on the lower right are 200 µm. **(E)** mRNA levels of EMT markers (E-cadherin, N-cadherin, Vimentin, α-SMA, Slug, Snail, Zeb1 and Zeb2) were detected in acidosis-adapted PDAC and their control cells by qPCR. **(F)** WB detected protein levels of Snail and Vimentin in acidosis-adapted PDAC and their control cells (the upper panel). Protein expression analysis of PANC-1 and SW1990 cells (the lower panel). **(G)** Immunofluorescence detection of E-cadherin and β-catenin in PANC-1-AA/NA and SW1990-AA/NA cells (red). Nuclei were stained in blue with DAPI. The white scale bars on the lower right in (A), (B), (C), (D) are 200 µm, in (G) is 75 µm. *,* P*<0.05; **,* P*<0.01; ***,* P*<0.001; ****, *P*<0.0001; ns, no significant difference; N=3.

We then tested the cellular behavior of acidosis-adapted cells. Wound healing test was used to detect the difference in migration ability between normally cultured PDAC cells and acidosis-adapted PDAC cells. We found that acidosis-adapted PDAC cells migrated faster than that of control cells. The healing rate of PANC-1-AA was about three times that of PANC-1-NA, while SW1990-AA was almost two times that of SW1990-NA (Fig. 2A & B). Transwell assays showed that, compared with PANC-1-NA cells, the number of invasive cells of PANC-1-AA was increased by about 50% (Fig. 2C). Similar to PANC-1 cells, the invasive cells of SW1990-AA increased 30% to SW1990-NA (Fig. 2D). As is well known, epithelial-mesenchymal transition (EMT) is an essential factor in promoting invasion and metastasis, so we detected the expression of EMT markers. As exhibited in Fig. 2E, mRNA levels of N-cadherin, Vimentin, α-SMA, Snail, Zeb1 and Zeb2 were significantly increased in PANC-1-AA and SW1990-AA cells. Snail, Vimentin and β-catenin protein levels increased as well (Fig. 2F & G), but E-cadherin protein level decreased in acidosis-adapted PDAC cells (Fig. 2G). Therefore, acidosis-adapted PDAC cells exhibited enhanced migration and invasion ability.

### Acidosis-adapted PDAC cells exhibited enhanced cell proliferation

**Figure 3:**
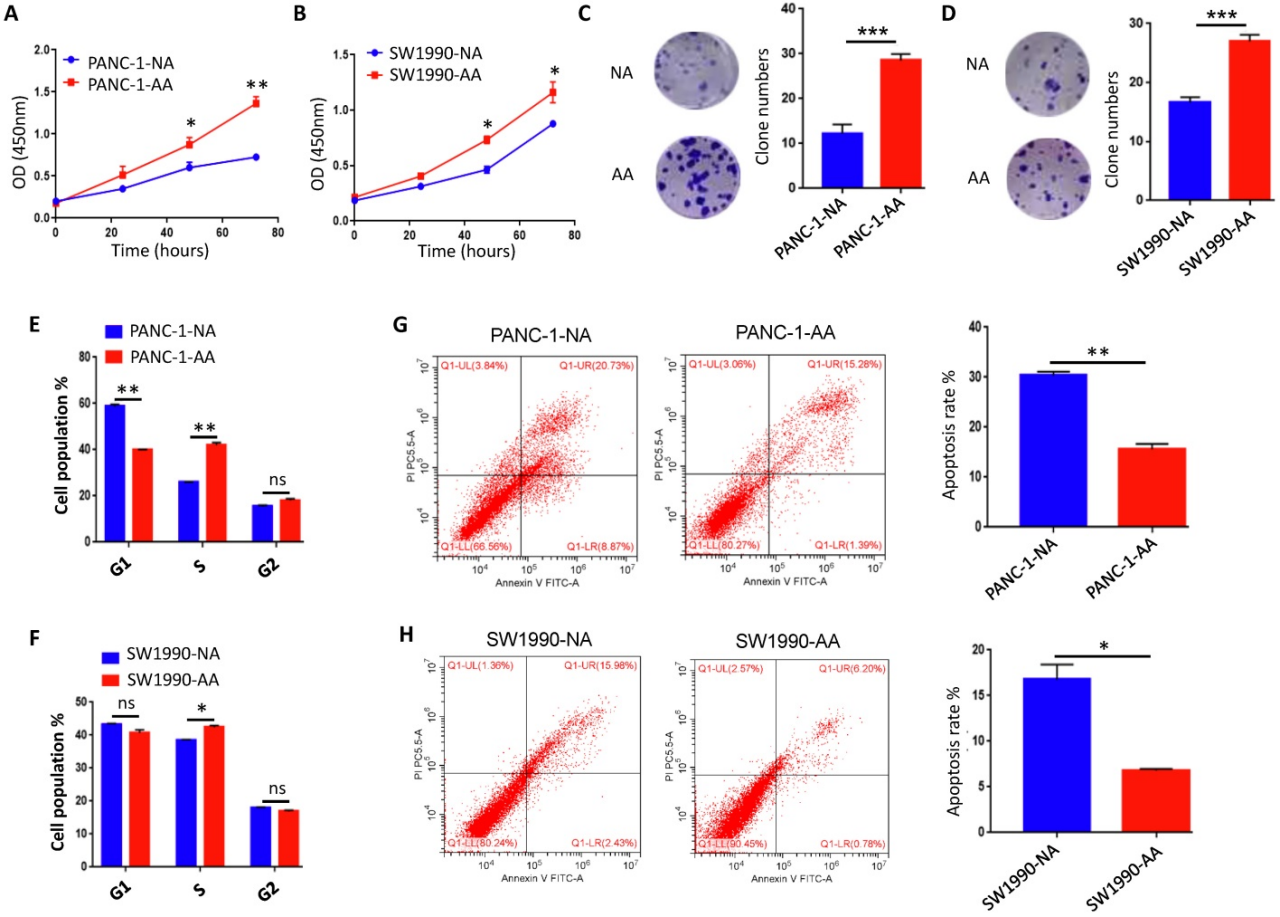
** Proliferation was increased in acidosis-adapted PDAC cell. (A & B)** CCK8 assays showed cell proliferation in PANC-1-AA/NA and SW1990-AA/NA cells. **(C & D)** Effects of acidic environment on the clonogenicity of PANC-1 and SW1990 cells. **(E & F)** Summary data of cell cycle analysis by flow cytometry in acidosis-adapted PDAC cells and control cells. **(G & H)** Cell apoptosis analysis by flow cytometry in acidosis-adapted PDAC cells and control cells (left) and summary data (right). *, P<0.05; **,* P*<0.01; ***,* P*<0.001; ns, no significant difference; N=3.

Previous studies revealed that acidosis-adapted colon cancer and cervical cancer cells could grow at a similar or higher rate in comparison to their parental cells 25. However, it is still unclear whether a chronic acidic microenvironment can affect the proliferation of PDAC cells. The results of CCK8 test showed that, compared with the NA cells in normal medium, the proliferation of PANC-1-AA and SW1990-AA cells were significantly increased in an acidic medium (Fig. 3A & B). The colony formation assay also reflected similar results (Fig. 3C & D). Furthermore, we compared the cell cycle of control cells and acidosis-adapted cells. The number of PANC-1-AA cells in S phase was significantly more than that in PANC-1-NA cells, but the number of G1 phase cells was markedly reduced. There was no significant difference in the number of G2 phase cells between NA and AA cells. In SW1990 cells, the number of acidosis-adapted cells in S phase was more than that in normal culture, but there was no significant difference in other phases (Fig. 3E & F). All cell cycle pictures of above cells are in Supplementary Fig. 2. On the contrary, the apoptosis of PANC-1-AA and SW1990-AA cells was less than that of PANC-1-NA and SW1990-NA cells (Fig. 3G & H). Therefore, acidosis-adapted cells seem to have enhanced proliferation and less apoptosis than parental cells.

### MMP1 was significantly upregulated in acidosis-adapted PDAC cells

**Figure 4:**
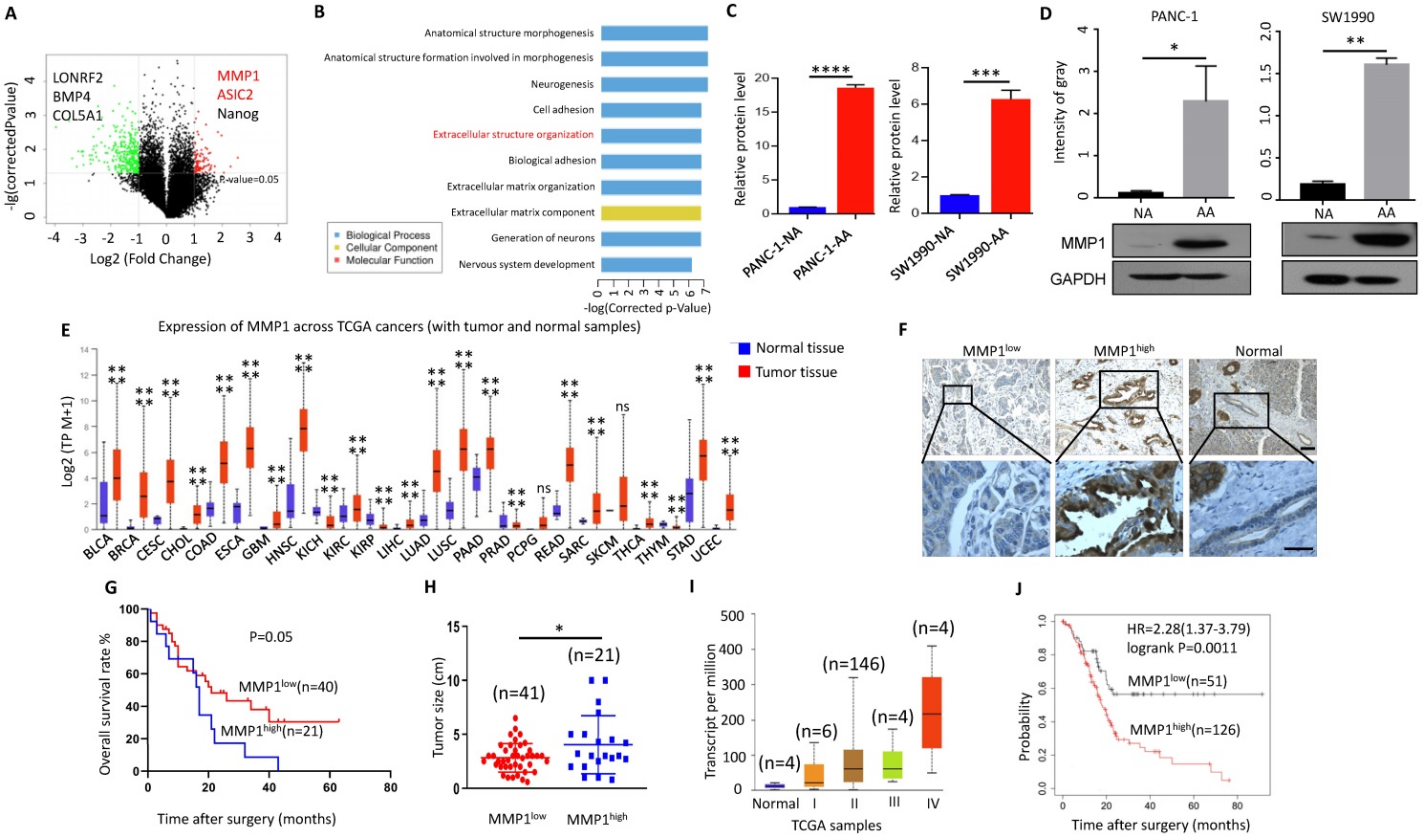
** MMP1 expression level was increased in acidosis-adapted PDAC cell. (A & B)** Results of high-throughput microarray profiling. **(C& D)** Transcription level and protein level of MMP1 were detected by qPCR and WB in acidosis-adapted PDAC cells and control cells. **(E)** Expression of MMP1 mRNA in various tumors from TCGA database. BLCA: bladder urothelial carcinoma; BRCA: breast invasive carcinoma; CESC: cervical squamous cell carcinoma; CHOL: cholangiocarcinoma; COAD: colon adenocarcinoma; ESCA: esophageal carcinoma; GBM: glioblastoma multiforme; HNSC: head and neck squamous cell carcinoma; KICH: kidney chromophobe; KIRC: kidney renal clear cell carcinoma; KIRP: kidney renal papillary cell carcinoma; LIHC: liver hepatocellular carcinoma; LUAD: lung adenocarcinoma; LUSC: lung squamous cell carcinoma; PAAD: pancreatic adenocarcinoma; PRAD: prostate adenocarcinoma; PCPG: pheochromocytoma and paraganglioma; READ: rectum adenocarcinoma; SARC: sarcoma; SKCM: skin cutaneous melanoma; THCA: thyroid carcinoma; THYM: thymoma; STAD: stomach adenocarcinoma; UCEC: uterine corpus endometrial carcinoma. **(F)** Representative images of immunohistological staining on MMP1 proteins in PDAC tissues (n=61 patients). The scale bars on the lower right are 200μm (the upper panel) and 100 µm (the lower panel). **(G)** Analysis of survival rates in PDAC patients with low and high MMP1 expression level (n=61 patients). **(H)** Relationship between the MMP1 protein expression level and the size of pancreatic cancer. **(I)** Expression of MMP1 in different tumor stages from TCGA database. **(J)** Analysis of prognosis of patients with high expression of MMP1 and low expression of MMP1 by Kaplan-Meier from KM plotter. *, P<0.05; **,* P*<0.01; ***,* P*<0.001; ****,* P*<0.0001; ns, no significant difference; N=3.

We analyzed the differentially expressed genes of PANC-1-NA and PANC-1-AA cells by microarray technology. We found that MMP1, ASIC2 and NANOG expressions were significantly upregulated in PANC-1-AA cells, while the expression of LONRF2, BMP4 and COL5A1 were markedly decreased. Among them, the most obviously changed gene was MMP1 (Fig. 4A). Interestingly, the glucose transporter SLC2A12 was also significantly upregulated in PANC-1-AA cells (Supplementary Table 2), implying the redirection of increased glucose from glycolysis to the PPP, which provides the substrates for nucleic acid, fatty acids, sterols, nucleotides and non-essential amino acids synthesis 23. GO analysis showed that the main difference between PANC-1-NA and PANC-1-AA cells were in cell adhesion and extracellular matrix organization (Fig. 4B). qPCR and WB assays showed that both mRNA and protein levels of MMP1 were increased either in PANC-1-AA cells or in SW1990-AA cells (Fig. 4C & D). The top 20 genes from the RNA-seq and the top 50 metabolites from metabonomic were listed in Supplementary Table 3.

MMP1 can degrade a variety of collagen and other extracellular matrix 26. MMP1 expression level is increased in various malignant tumors and is correlated with poor prognosis 27. TCGA database showed that the expression of MMP1 in most cancer tissues was significantly higher than that in adjacent tissues (Fig. 4E). However, the relationship between MMP1 and pancreatic cancer is rarely studied, especially in the acidic microenvironment of pancreatic cancer. We detected the expression of MMP1 in tissues of 61 patients with PDAC via immunohistochemistry (Fig. 4F), and found that higher the expression of MMP1 was associated with worse prognosis (Fig. 4G). The tumor size was larger in the MMP1high group than MMP1low (Fig. 4H). Consistently, bioinformatic analysis revealed that the expression level of MMP1 was correlated with advanced tumor stage (Fig. 4I) and poor prognosis (Fig. 4J). Pathological characteristics of clinical samples revealed that high expression of MMP1 is associated with metastasis and acidic marker, carbonic anhydrase 9 (CA9), and acid-sensing ion channels, ASIC1 and ASIC2 (Table 1). These results showed that elevated MMP1 expression might at least partially mediate the enhanced invasion capacity of acidosis-adapted PDAC cells.

### Knock-down of MMP1 impaired invasion and metastasis of acidosis-adapted PDAC cells

**Figure 5:**
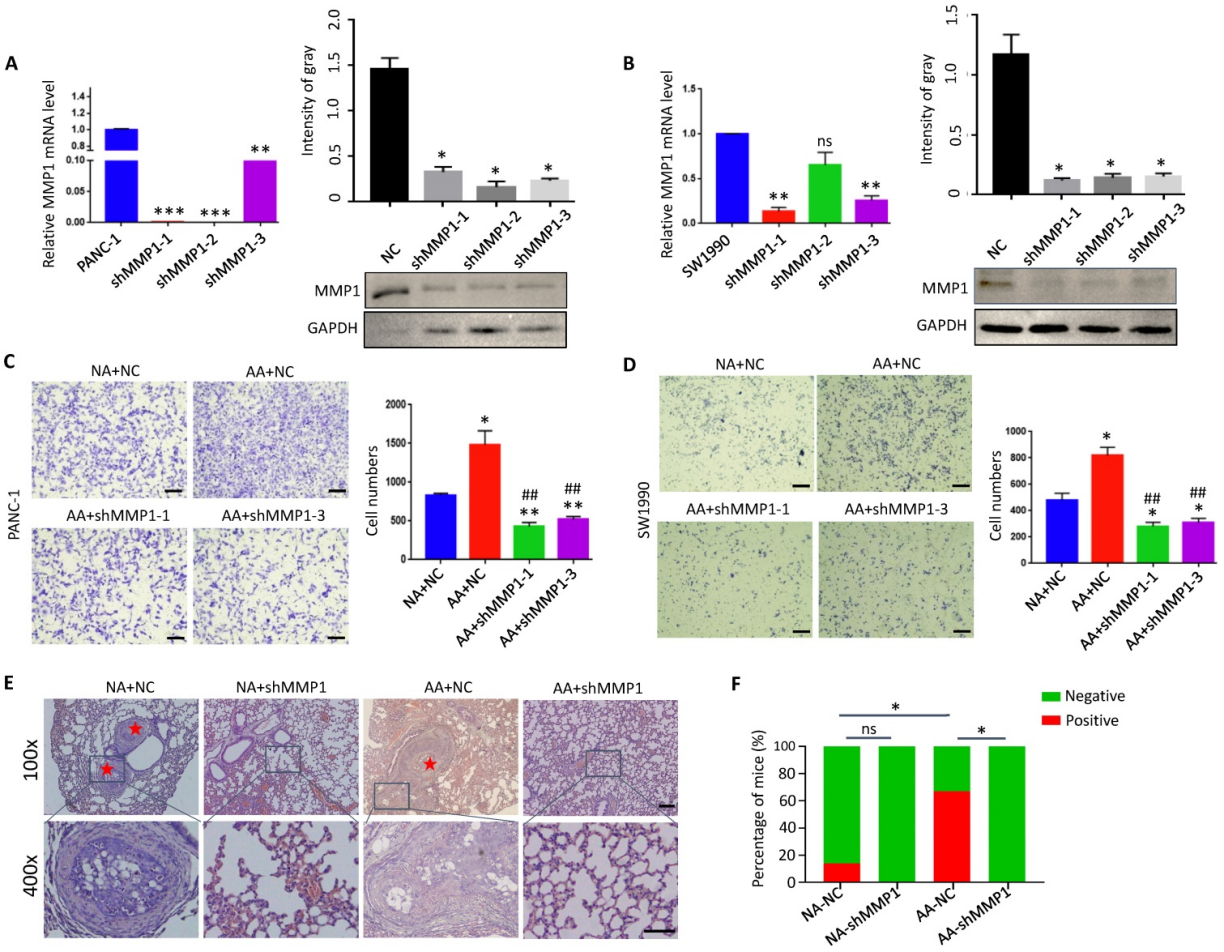
** Knockdown of MMP1 impaired acidosis-adapted PDAC cell invasion. (A & B)** mRNA levels (left) and protein levels (right) of MMP1 detected by qPCR and WB in MMP1-knockdown PANC-1 and SW1990 cells. **(C & D)** PDAC cell invasion presented as original images and presented as summary data in MMP1-knockdown PANC-1-AA and SW1990-AA cells. The scale bars on the lower right are 200 µm. **(E)** Representative images of H&E staining of tail vein injection models. Scale bars: 100× magnification, 200 µm; 400× magnification, 50 µm. **(F)** Summary data of tumor numbers in tail vein injection model. *, P<0.05; **, P<0.01; ***, P<0.001; vs NC*; ##, P*<0.01 vs AA; ns, no significant difference; N=3.

Database analysis (https://hgserver1.amc.nl) showed that the expression of MMP1 was positively correlated with acidic markers such as CA9 (*P*<0.0001), MCT1 (monocarboxylate transporter 1) (*P*<0.0001) and MCT4 (monocarboxylate transporter 4) (*P*=0.0004), and Lamp2 (Recombinant Lysosomal Associated Membrane Protein 2) to a lesser degree (*P*=0.059) in PDAC tissues (Supplementary Fig. 3). These results suggest that MMP1 is closely related to the acidic microenvironment. We knocked down the expression of MMP1 by interfering with lentivirus in PANC-1 and SW1990 cells (Fig. 5A & B), and found that the invasion ability of acidosis-adapted cells was significantly reduced even in an acidic environment, and the number of invasive cells decreased significantly compared with normal cultured cells (Fig. 5C & D). The *in vivo* impact of MMP1 was evaluated with the tail vein injection model. The results showed an enhanced metastatic potential in acidosis-adapted PDAC cells compared to parental cells, and knocking down MMP1 expression significantly decreased *in vivo* metastasis of AA cells (Fig. 5E & F). Thus, MMP1 plays a critical role in mediating the enhanced invasion and metastasis of acidosis-adapted PDAC cells.

### Activation of YAP/TAZ signaling upregulated MMP1 in acidosis-adapted PDAC cells

**Figure 6:**
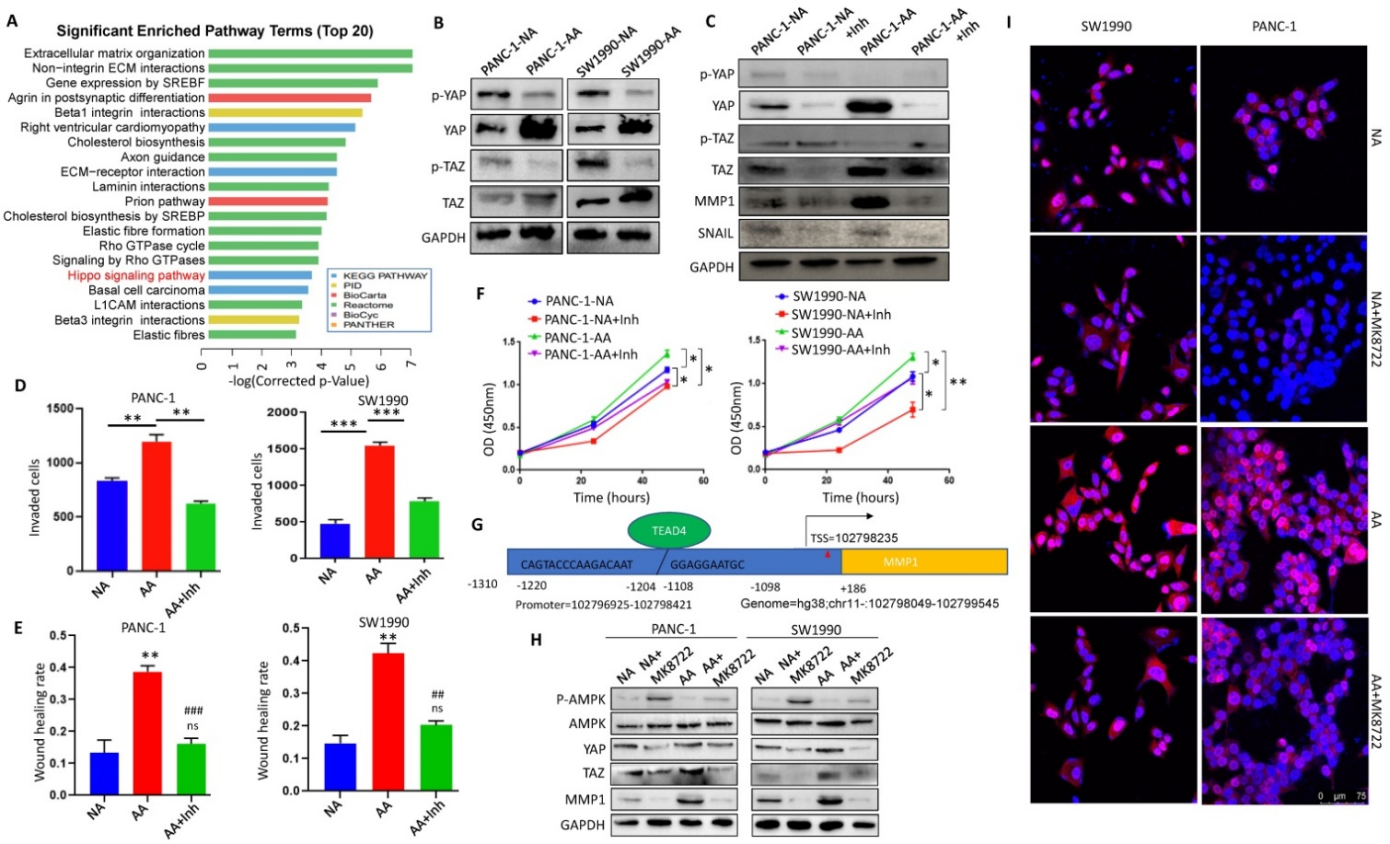
** Inactivation of Hippo pathway mediated MMP1 overexpression in acidosis-adapted cells. (A)** KEGG analysis of the differentially expressed genes showed that the Hippo pathway was significantly changed in acidosis-adapted PDAC cells. **(B)** Protein levels of YAP and TAZ in acidosis-adapted PDAC cells and control cells were detected by WB. **(C)** The protein levels of YAP, MMP1 and Snail were detected by WB after treated with 10 µM YAP/TAZ inhibitor ML-7 for 24h in PANC-1 and SW1990. **(D)** Summary data of transwell after treated with YAP/TAZ inhibitor ML-7 for 24h in PANC-1 and SW1990. **(E)** Summary data of wound healing assays after treatment with ML-7 for 24h in PANC-1 and SW1990 cells. **(F)** Cell proliferation was detected by CCK8 after treatment with ML-7. **(G)** Binding sites prediction between TEADs and MMP1 promoter. **(H)** WB detection of p-AMPK, AMPK, YAP, MMP1 protein expression after treated with AMPK inhibitor MK8722 at 1 µM for 24 h in PANC-1 and SW1990 cells. **(I)** The expression and nuclear translocation of YAP were detected by immunofluorescence. Scale bar is 75 µm. *, P<0.05; **,* P*<0.01; ***, P<0.001 vs NC;* ##, P*<0.01,* ###, P*<0.001 vs AA; ns, no significance difference; N=3.

In order to explore the regulatory mechanism of the acidic microenvironment of MMP1 in PDAC cells, we reviewed the microarray results and found that the Hippo signaling pathway was significantly changed (Fig. 6A). We detected the expression and activation of YAP and TAZ, the key molecules of Hippo pathway. The results showed that expression levels of YAP and TAZ in PANC-1-AA and SW1990-AA cells were significantly higher than those in PANC-1-NA and SW1990-NA cells (Fig. 6B). Protein expression analysis was in Supplementary Fig. 4A. When YAP/TAZ is phosphorylated, their activities are inhibited. Non-phosphorylated YAP/TAZ will enter the nucleus as co-transcription factors and combine with TEA domain transcription factors (TEADs) to promote oncogene transcription 28. Thus, we chose YAP/TAZ inhibitor ML-7 to block their activities. After treatment with ML-7, the activation level of YAP and TAZ were significantly reduced, while the expression of MMP1 and Snail in PANC-1-AA and SW1990-AA cells were significantly decreased (Fig. 6C). Protein expression analysis was in Supplementary Fig. 4B. Transwell assays showed that the invasion capacity of PANC-1-AA and SW1990-AA cells was reduced by about 50% after being treated with ML-7 (Fig. 6D). The results of the scratch test were consistent with those of transwell (Fig. 6E). Transwell and wound healing images are shown in Supplementary Fig. 5. In addition, YAP/TAZ inhibitor also significantly inhibited the proliferation of acidosis-adapted cells, compared with NA cells (Fig. 6F). Moreover, we found a positive correlation between the expression of YAP and MMP1 in PDAC tissues (Supplementary Fig. 6). There is a predicted binding site of TEAD4 in the promoter region of MMP1, which is a recognized YAP/TAZ effector molecule 29 (Fig. 6G). We treated PDAC cells with different concentrations of AMPK activator, MK8722, and screened the optimal working concentration to be 1 μM (Supplementary Fig. 7A). MK8722 treatment could dramatically downregulate both YAP and MMP1 (Fig. 6H & Supplementary Fig. 7B). Besides, we found that more nucleus-located YAP in acidosis-adapted cells, which can be decreased by MK8722 (Fig. 6I). These results indicated that the activation of AMPK mediated the decreased MMP1 expression through restraining Hippo signaling.

## Discussion

Studies have shown that tumor cells prefer forming an acidic microenvironment because of high metabolic activity and insufficient perfusion. Tumor cells that adapt to an acidic microenvironment usually exhibit aggressive phenotypes, which is achieved by acid-induced cell motility, extracellular matrix degradation, decreased immune responses and modified signaling pathways 30. However, its specific regulatory mechanism has not been fully clarified. Our experiments elucidated that glycolysis was significantly inhibited, while intracellular ATP, glucose and other metabolites were increased, in acidosis-adapted PDAC cells. This metabolic reprogramming leads to AMPK inactivation. It should be noted that the glucose transporter SLC2A12 (GLUT12) rather than SLC2A1 (GLUT1) was also upregulated in acidosis-adapted PDAC cells. GLUT12 was required for maximal androgen-mediated glucose uptake and cell growth in prostate cancer cells (LNCaP and VCaP) 31. In addition, GLUT12 is highly expressed in triple-negative breast cancer (TNBC) samples and promotes TNBC tumor growth and metastasis *in vitro* and *in vivo* by regulating aerobic glycolysis 32. This evidence implies that acidosis-adapted uptake more glucose to fuel their aggressive activities.

Although the intracellular glucose was elevated in acidosis-adapted PDAC cells, it was not used for glycolysis due to the many intermediate products of glycolysis were reduced. PPP is the other way for glucose catabolism. The PPP branches after the first step of glycolysis and consumes the intermediate G6P to generate F6P and glyceraldehyde 3-phosphate (G3P). The PPP supplies NADPH and R5P instead of ATP 23. These two metabolites are vital for cell survival and proliferation. R5P is a building block for nucleic acid synthesis. NADPH is the reducing power required to synthesize fatty acids, sterols, nucleotides and non-essential amino acids 24. Moreover, NADPH mediates oxidized glutathione (GSSG) conversion to reduced glutathione (GSH) via glutathione reductase to reduce the reactive oxygen species. Recently, the PPP has been reported to enhance the chemoresistance of breast cancer cells 33. We have reasons to propose that the glucose was re-directed to PPP as the increase in intracellular glucose, amino acids, phosphatidylcholine, phosphosphingolipids, and the reduction in glycolysis intermediates. Consistently, we found that the activity of G6PDH, a PPP key enzyme, increased significantly in acidosis-adapted cells, and the ratio of NADP/NADPH decreased. However, the mRNA levels of the PPP enzymes were not altered in the acidosis-adapted PDAC cells (data not shown). This result indicates that there might be post-transcriptional regulation of this pathway.

**Figure 7:**
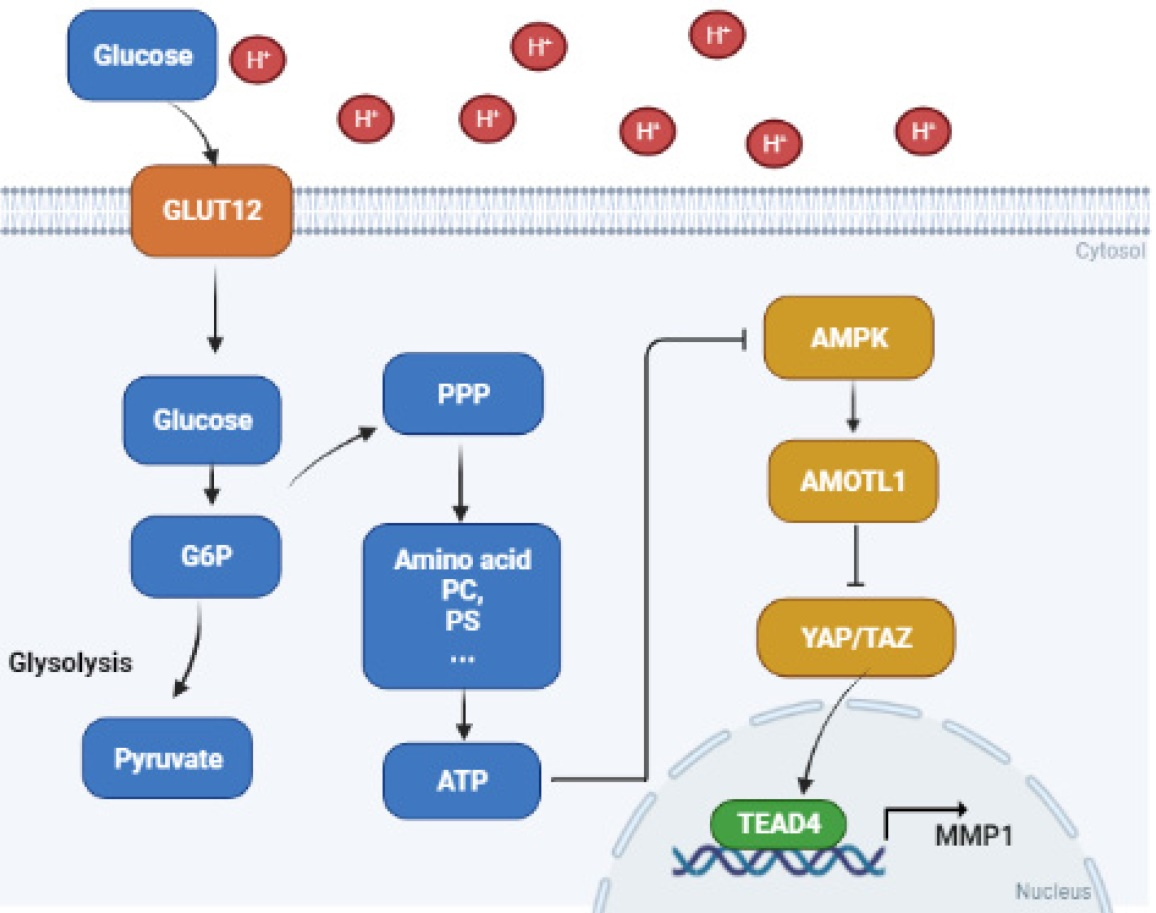
** Proposed mechanisms of acidosis promoted PDAC progression.** In acidosis-adapted PDAC cells, glucose enters the cells through GLUT12 transporter, which increases glucose-6-phosphate (G6P) and pentose phosphate pathway (PPP) metabolism, thus affecting amino acids or phosphatidylcholine (PC), phosphosphingolipids (PS) and other metabolism, increased ATP content and inhibited AMPK, so as to activate YAP/TAZ through AMOTL1. YAP/TAZ translocated into the nucleus and bound with TEAD4 to promote MMP1 transcription and increase expression level of MMP1, leading to PDAC cell metastasis and proliferation.

The increased ATP thus inactivated the AMPK pathway, which is generally considered a metabolic sensor to suppress cancer. AMPK could initiate a series of downstream pathways, including Hippo. We found that the Hippo pathway effector molecules YAP and TAZ were significantly activated in acidosis-adapted PDAC cells. Then, YAP/TAZ bound to transcription factor TEAD4 in nucleus, and facilitated MMP1 expression, resulting in PDAC progression (Fig. 7). Previous studies demonstrated that other MMPs are regulated by an acidic condition in different diseases. The expression levels of MMP3 and MMP13 were elevated under the degenerated intervertebral disc-like acidic condition 34. MMP12 is also increased by extracellular acidosis in systemic sclerosis 35. An acidic microenvironment induces MMP2, MMP3, MMP9 and MMP13 expression to promote breast or lung cancer progression 36. MMP1 belongs to the zinc-dependent endopeptidase family. It has been shown to be closely relevant to proliferation, migration and invasion in various cancers 37. MMP1 can also promote the development of pancreatic cancer 38, even become a potential diagnostic marker for pancreatic cancer 39. However, there is less research on the regulation of MMP1 in the cancer acidic microenvironment. Our study revealed that the expression of MMP1 was increased by acidosis, enriching the research on the regulation of the acidic microenvironment on the MMP family.

At present, many studies have found that MMP1 can promote cell proliferation. Marta Gabasa et al. found MMP1 could promote large cell carcinoma of the lung by combining with TGF-β1 to induce paracrine fibroblast senescence 40. MMP1 promotes colorectal cancer 41 and esophageal squamous cell carcinoma 42 proliferation by activating PI3K-AKT signaling pathway. MMP1 also enhances liver cancer 37 and endometrial cancer 43 proliferation. There are few studies on the regulation of PDAC by MMP1, however, the specific mechanism is unclear. We found that MMP1 can also promote PDAC cells proliferation during acidosis environment, the downstream pathway of MMP1 will be further studied in our next experimental plan.

## Conclusion

In summary, the present study showed that metabolic reprogramming towards PPP promotes metastasis and proliferation of acidosis-adapted PDAC cells via AMPK/YAP/MMP1 axis, enriching the mechanism by which acidic environment promotes tumor progression.

## Supplementary Material

Supplementary materials and methods, figures and tables.

## Figures and Tables

**Table 1:** The correlation between MMP1 expression level and clinicopathological parameters

Features	No. of patients (%)	MMP1 expression status		*P*
		Low (n=41) No. patient (%)	High (n=21) No. patient (%)	
**Gender**				0.410
Male	24(38.7)	14(58.3)	10(41.7)	
Female	38(61.3)	27(71.1)	11(28.9)	
**Age**				0.590
≤59	34(54.8)	21(61.8)	13(38.2)	
>59	28(45.2)	20(71.4)	8(28.6)	
**T stage**				
1+2	47(75.8)	35(74.5)	12(25.5)	**0.026**
3+4	15(24.2)	6(40.0)	9(60.0)	
**N stage**				0.095
0	42(67.7)	27(64.3)	15(35.7)	
1	16(25.8)	13(81.2)	3(18.8)	
2	4(6.5)	1(25.0)	3(75.0)	
**M stage**				
0	61(98.4)	40(65.6)	21(34.4)	0.471
1	1(1.6)	1(100)	0(0)	
**TNM stage**				
I	31(50.0)	24(77.4)	7(22.6)	0.213
II	26(41.9)	14(53.8)	12(46.2)	
III	4(6.5)	2(50.0)	2(50.0)	
IV	1(1.6)	1(100)	0(0)	
**Vessel invasion**				0.414
without	34(54.8)	24(70.6)	10(29.4)	
with	28(45.2)	17(60.7)	11(39.3)	
**Nerve invasion**				**0.040**
without	29(46.8)	23(79.3)	6(20.7)	
with	33(53.2)	18(54.5)	15(45.5)	
**Differentiation degree**				0.393
Poor	20(32.3)	13(65.0)	7(35.0)	
Moderate	27(43.5)	20(74.0)	7(26.0)	
Well	15(24.2)	8(53.3)	7(46.7)	
**CA9 expression**				**0.018**
low	43(69.4)	33(76.7)	10(23.3)	
high	19(30.6)	8(42.1)	11(57.9)	
**ASIC1 expression**				**0.013**
low	25(40.3)	21(84.0)	4(16.0)	
high	37(59.7)	20(54.1)	17(45.9)	
**ASIC2 expression**				**0.010**
low	41(66.1)	32(78.0)	9(22.0)	
high	21(33.9)	9(42.9)	12(57.1)	

61 PDAC clinical samples were used to evaluate the correlation between MMP1 expression and clinicopathological features. The bold values indicated that the P value was smaller than 0.05.

## Abbreviations

PDAC: pancreatic ductal adenocarcinoma
PPP: pentose phosphate pathway
EMT: epithelial mesenchymal transition
MMP1: matrix metalloproteinase 1
MST1/2: Ste20-like kinases 1/2
LATS1/2: large tumor suppressor 1/2
YAP: yes association protein
TEAD: TEA domain
CA9: Carbonic Anhydrase 9
MCT1: monocarboxylate transporter 1
MCT4: monocarboxylate transporter 4
Lamp2: Recombinant Lysosomal Associated Membrane Protein 2
AMP: adenosine monophosphate
F6P: fructose-6-phosphate
F1P: fructose-1-phosphate
FBP: fructose-1,6-bisphosphate
G6P: glucose-6-phosphate
G6PDH: glucose-6-phosphate dehydrogenase
NADPH: reduced nicotinamide adenine dinucleotide phosphate
ROS: reactive oxygen species
PC: phosphatidylcholine
PS: phosphosphingolipids
