# Supplementary material for: Enhanced pentose phosphate pathway activity promotes pancreatic ductal adenocarcinoma progression via activating YAP/MMP1 axis under chronic acidosis
Source: Int J Biol Sci. 2022 Mar 6;18(6):2304–16. doi: 10.7150/ijbs.69526 (PMC8990471; doi:10.7150/ijbs.69526)
Supplement: Supplementary file 1 — Supplementary materials and methods, figures and tables. [file ijbsv18p2304s1.pdf]

Supplementary Materials and Methods

RNA extraction and real-time quantitative PCR (qPCR)

Total RNA was extracted from each group with TRIzol Reagent (Takara, Dalian, China). The cDNA was obtained by using PrimeScript RT Master Mix (Takara, Japan). 50ng of each cDNA was amplified as a template, and qPCR was performed in a CFX96 real-time PCR system (Bio-Rad, Hercules, CA, USA) with SYBR® Premix Ex TaqTM II kit (Takara, Japan). The PCR primers used are listed in Supplementary Table 1.

Wound healing assay

After cell fusion rate in the 6-well plate reached more than 90%, the cell medium was removed. A 10μL pipette tip was used to draw a straight line in the cells along the sterilized ruler. Cells were washed with PBS 3 times. Cell migration was observed and recorded at 0 h and 24 h.

Cell migration and invasion assays

Twenty-four-well transwell chambers were used for this assay (BD Falcon, Franklin Lakes, NJ). Briefly, 3×10^4 cells were plated into each upper chamber with 8-μm pores and cultured in 200μL serum-free DMEM. The lower chambers were filled with 500μL complete DMEM. After incubation for 24 hours at 37°C, cells that had migrated in the lower chambers were fixed with 4% polyformaldehyde and stained with 0.1% crystal violet. The number of cells was counted in 5 distinct areas at ×100 magnification. The results represent the average cell number in 3 wells per cell line. Then, the upper surface of the polycarbonate filter was coated with 10% Matrigel TM (BD Biosciences, Franklin Lakes, NJ), and 5×10^4 cells were added to detect cell invasion. The other conditions were the same as those in the migration assay.

Immunofluorescence assay

Cells were fixed with 4% polyformaldehyde for 20 min, and blocked with goat serum for 1 h at room temperature. Cells were then incubated with an anti-E-cadherin or β-actin antibody for 1 h at room temperature. After three washes in PBS, cells were incubated with a Cy3-conjugated secondary antibody (Beyotime, China) for 1 h. The nuclei were stained with DAPI for 5 min. Images were captured on a confocal

microscope (Leica SP5, Germany).

Cell proliferation assay

Cells were plated in 96-well plates (2000 cells/well) in triplicate and measured using Cell Counting Kit-8 (CCK-8) (Dojindo Laboratories, Kumamoto, Japan). Cell proliferation was determined every 24 h for three days following the manufacturer's protocol. The absorbance at 450 nm was measured with a microplate reader (Bio-Rad, Hercules, CA, USA).

Colony formation assay

Long-term survival of cells was assessed by their ability to form colonies. 100 cells were seeded in a 96-well plate per well. After 15 days, colonies were fixed and stained with 0.1% crystal violate (Invitrogen, Carlsbad, CA, USA) before counting.

Cell cycle analysis

For cell cycle detection, 1.0×10^6 cells were harvested and fixed with 75% ethanol at 4°C overnight after being washed with PBS. Then, cells were washed with PBS and stained with propidium iodide. FACSCalibur instrument (BD Biosciences, Franklin Lakes, NJ, USA) and CellQuest software were used to analyze the cell cycle.

Cell apoptosis assay

Cells were obtained at a density of 1.0×10^6 cells/mL and washed with PBS, then incubated with reagents from the Annexin-V-FITC Apoptosis Detection Kit (Neobioscience, Shenzhen, China) according to the manufacturer's protocol. Then the cells were analyzed by FACS Vantage SE flow cytometer (BD Biosciences, San Jose, CA, USA).

Immunohistochemistry (IHC)

Following deparaffinization and rehydration, tissue samples (5mm slices) were incubated in 0.3% H_2O_2 in methanol for 30 min at 37 °C to block endogenous peroxidase. The sections were then boiled in 10 mmol/L citrate buffer (pH 6.0) for 2 min in an autoclave. Tissue samples were incubated with anti-MMP1 (Abcam, UK) overnight at 4 °C. MMP1 was detected using HRP-conjugated anti-rabbit secondary antibody (ZSGB-BIO, China) and visualized with DAB. The negative control was only

incubated with the secondary antibody. The intensity of staining (brown color) was semi-quantitatively scored as follows: 1, weak; 2, medium; 3, strong; and 4, very strong. The percentage of maximally stained tumor cells in each section was recorded (0, <5%; 1, 5–30%; 2, 30–50%; 3, >50%). High expression of MMP1 was defined as a combined score of the intensity and area of staining that was larger than 6. The results were verified by two pathologists independently.

Western blotting

Whole-cell lysates were extracted in a lysis buffer (Beyotime, Shanghai, China) and were separated by 10% SDS-PAGE, then transferred to polyvinylidene fluoride membranes (Millipore, Billerica, MA, USA) by using a wet transfer apparatus (Bio-Rad, German). Blots were blocked in 5% (w/v) skim milk for 2 h at room temperature and then incubated with antibodies against snail, Vimentin, MMP1 (Abcam, Cambridge, UK), P-TAZ, P-YAP, TAZ, YAP, AMPK, p-AMPK (Cell Signaling Technology, USA) and GAPDH (ZSGB-BIO, China) overnight at 4 °C. The HRP-coupled anti-rabbit secondary antibody (ZSGB-BIO, China) was used at a final dilution of 1:1000, visualized with an enhanced chemiluminescence (ECL) detection system (Thermo Scientific, Waltham, MA, USA).

Supplementary Figures:

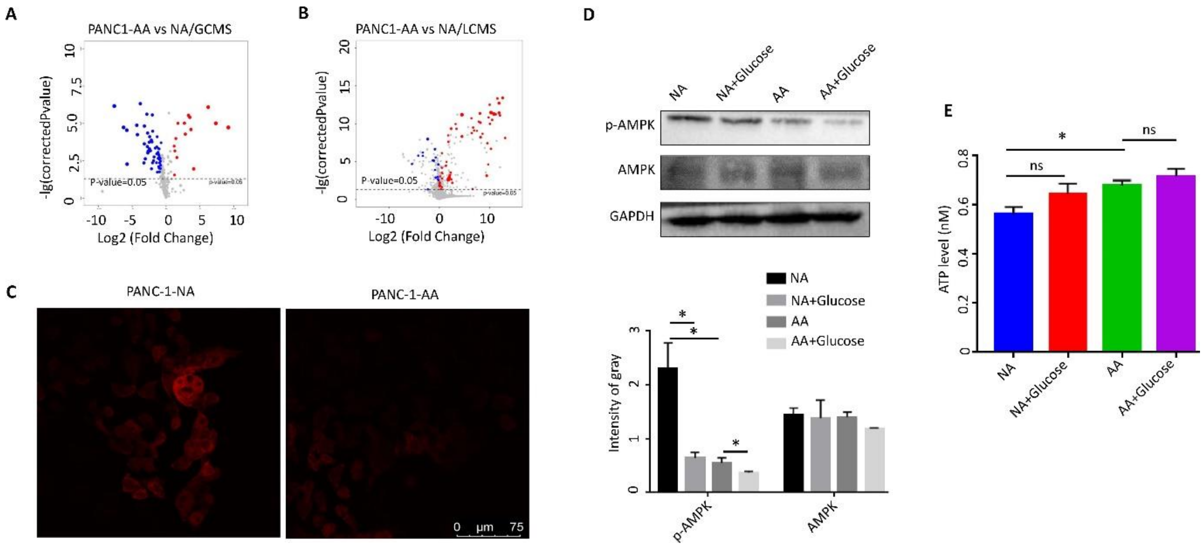

Supplementary Fig 1. Changes of metabolites in acidosis-adapted PANC-1 cells.

(A) The GC-MS detected changes of metabolites in PANC-1-NA and PANC-1-AA cells. **(B)** The LC-MS revealed changes of metabolites in PANC-1-NA and PANC-1-AA cells. **(C)** The content of ROS in PANC-1-NA and PANC-1-AA was detected by probe. The scale bar is 75 μm. **(D)** The effect of glucose on AMPK activation was detected by WB in SW1990-NA and SW1990-AA. **(E)** The effect of glucose on ATP content was detected in SW1990-NA and SW1990-AA. **, P<0.05*; ns, no significance difference; N=3.

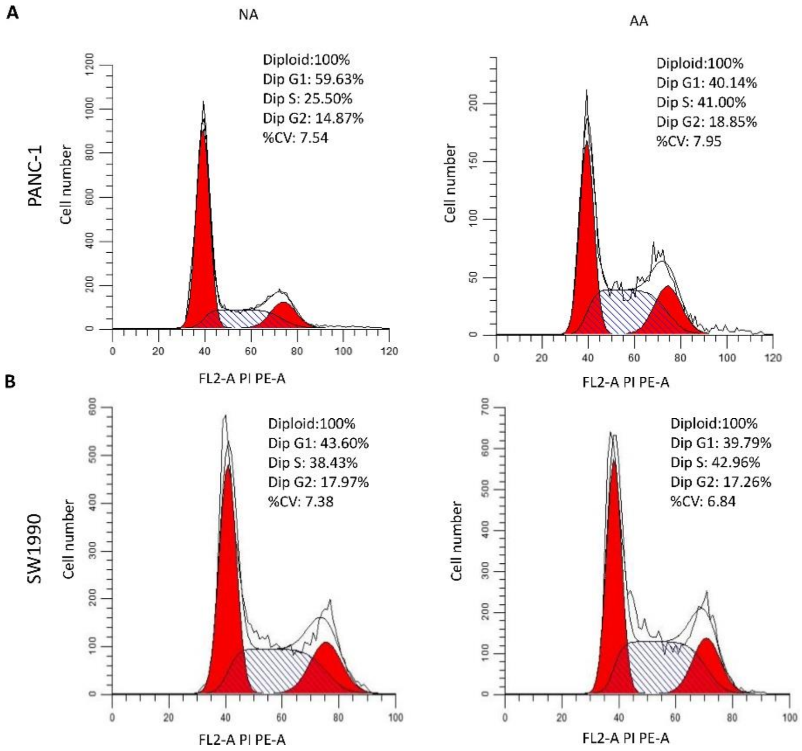

Supplementary Fig 2. Flow cytometry of cell cycle.

(A) Flow cytometry images of PANC-1-AA/NA cells. **(B)** Flow cytometry images of SW1990-AA/NA cells.

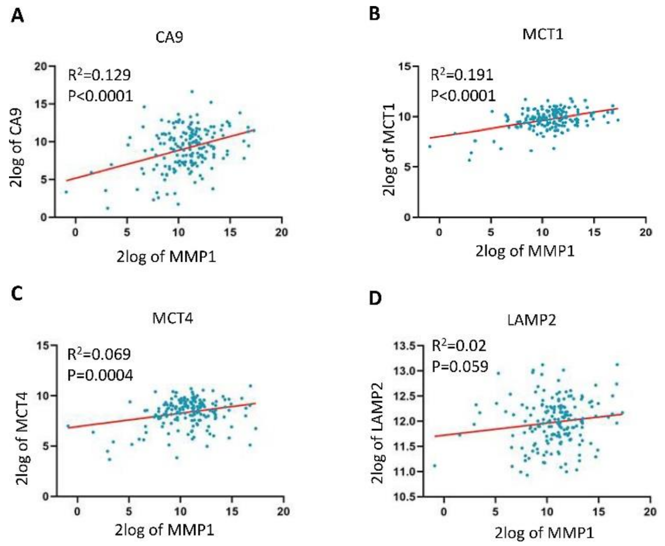

Supplementary Fig 3. Correlation between MMP1 expression and acidic markers.

(A-D) Database analysis (https://hgserver1.amc.nl) showed the relationship between MMP1 and CA9, MCT1, MCT4 and LAMP2.

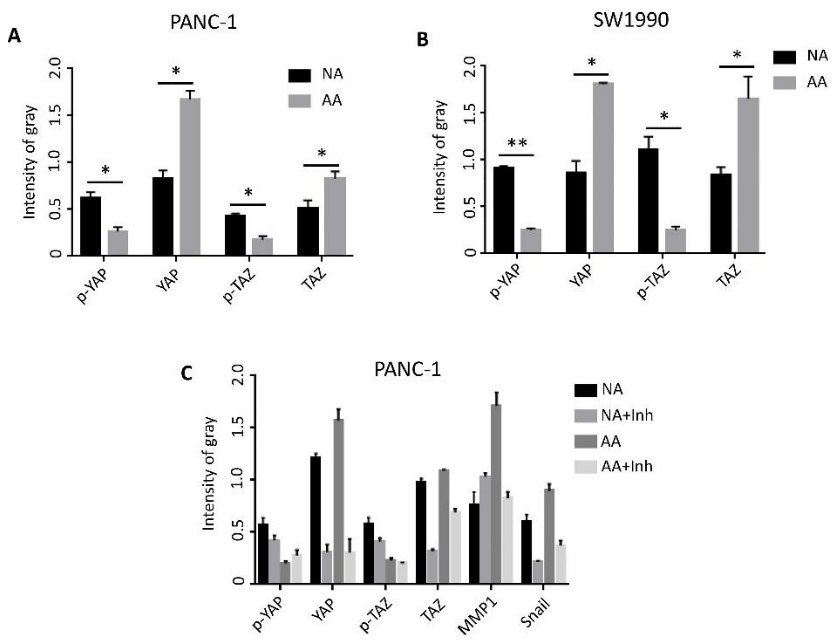

Supplementary Fig 4. Effect of acidosis microenvironment on Hippo pathway and its downstream MMP1.

(A)&(B) Statistics of protein expression of YAP, p-YAP, TAZ and p-TAZ in acidosis-adapted PDAC cells and control cells. **(C)** Statistics of protein expression of YAP, p-YAP, TAZ, p-TAZ, MMP1, Snail in PANC-1-NA and PANC-1-AA cells after treated with ML-7 for 24h. **, P<0.05*; ***, P<0.01*; N=3.

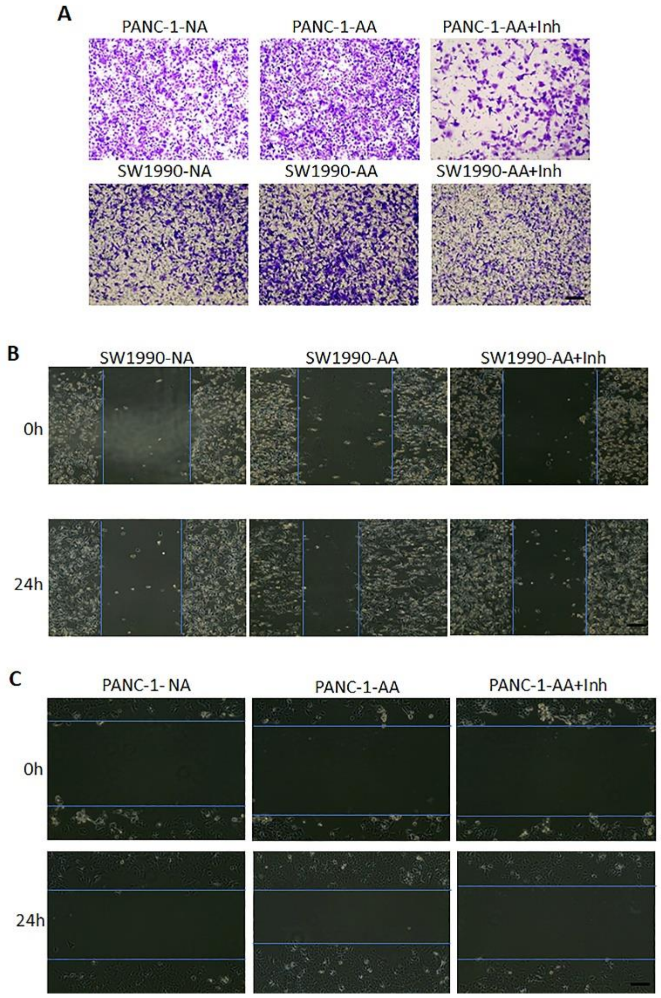

Supplementary Fig 5. Effect of YAP on invasion and migration of PDAC.

(A) Images of transwell in PANC-1-AA/NA and SW1990-AA/NA cells after treatment with ML-7. **(B)&(C)** Images of wound healing in PANC-1-AA/NA and SW1990-AA/NA cells after treatment with ML-7. The scale bars on the lower right are 200μm.

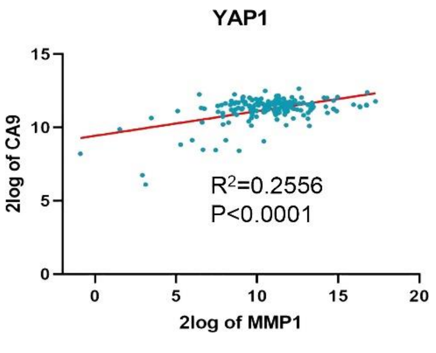

Supplementary Fig 6. Correlation analysis of YAP and MMP1 expression.

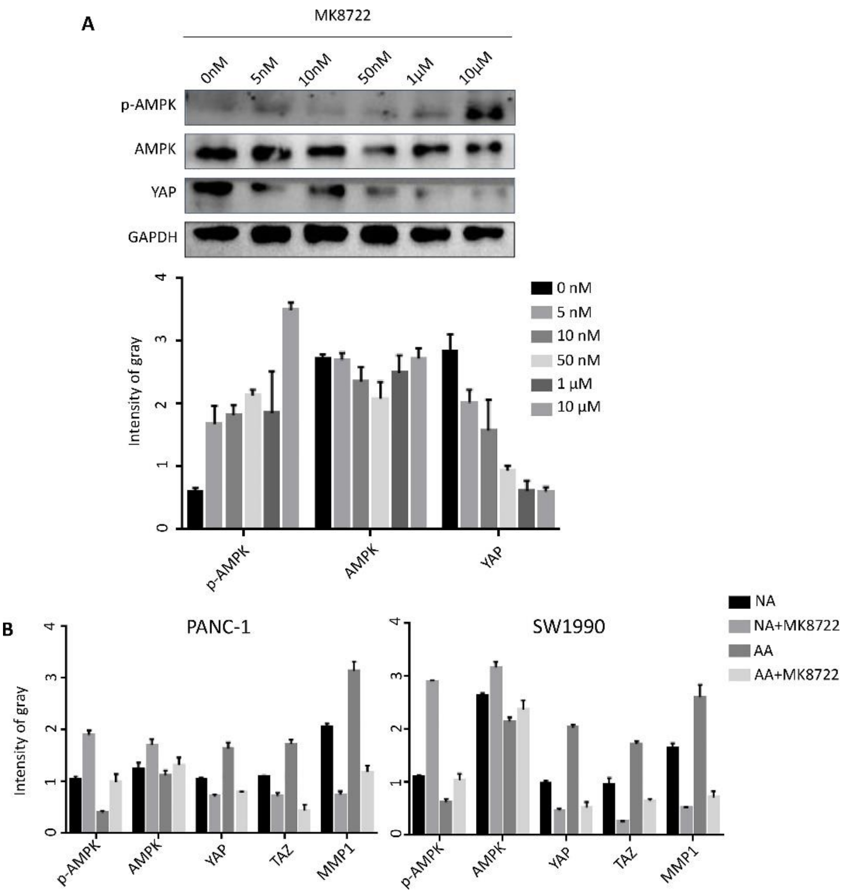

Supplementary Fig 7. Effect of AMPK inhibitor on Hippo pathway. (A) After the cells were treated with AMPK inhibitor MK8722 10nM for 24 hours, the activation of p-AMPK was detected by WB (the upper panel). The summary data was in the lower panel. **(B)** The analysis of activation of AMPK, Hippo and the expression of MMP1 in PDAC cells after treated with 10nM MK8722 for 24h.

Supplementary Tables:

Supplementary Table 1. Primers used in the present study

Primers	Sequence
E-cadherin	5′-AGTCACTGACACCAACGATAAT-3′(forward)
	5′-ATCGTTGTTCACTGGATTTGTG-3′(reverse)
N-cadherin	5′- CGATAAGGATCAACCCCATACA-3′(forward)
	5′-TTCAAAGTCGATTGGTTTGACC-3′(reverse)
Vimentin	5′- CCTTCGTGAATACCAAGACCTGCTC-3′(forward)
	5′- AATCCTGCTCTCCTCGCCTTCC-3′(reverse)
α-SMA	5′- CTTCGTTACTACTGCTGAGCGTGAG-3′(forward)
	5′- CCCATCAGGCAACTCGTAACTCTTC-3′(reverse)
Slug	5′- CTGTGACAAGGAATATGTGAGC-3′(forward)
	5′-CTAATGTGTCCTTGAAGCAACC-3′(reverse)
Snail	5′- CCTCGCTGCCAATGCTCATCTG-3′(forward)
	5′- AGCCTTTCCCACTGTCCTCATCTG-3′(reverse)
Zeb1	5′-CAGGCAAAGTAAATATCCCTGC-3′(forward)
	5′-GGTAAAACTGGGGAGTTAGTCA-3′(reverse)
Zeb2	5′-GAAGACAGAGAGTGGCATGTAT-3′(forward)
	5′- GTGTGTTCGTATTTATGTCGCA-3′(reverse)
MMP1	5′-AGATTCTACATGCGCACAAATC-3′ (forward)
	5′-CCTTTGAAAAACCGGACTTCAT-3′ (reverse)
GAPDH	5'- GCACCGTCAAGGCTGAGAAC -3'(forward)
	5'- TGGTGAAGACGCCAGTGGA -3' (reverse)

The above primers were used for real-time quantitative PCR experiment.

Supplementary Table 2. The fold change of glucose transporters in acidosis-adapted PANC1 cells

Gene	Family group	Fold change	Trend	P value
SLC2A12	GLUT	4.50	up	0.01
SLC2A11	GLUT	1.42	up	0.07
SLC2A1	GLUT	1.32	down	0.01
SLC2A14	GLUT	1.25	down	0.04
SLC2A5	GLUT	1.01	down	0.84
SLC2A8	GLUT	2.22	down	0.01
SLC2A6	GLUT	1.22	down	0.11
SLC2A9	GLUT	1.43	down	0.20
SLC5A12	SGLT	1.13	up	0.05
SLC5A5	SGLT	1.11	down	0.23
SLC5A3	SGLT	1.00	up	0.91
SLC5A6	SGLT	1.15	up	0.45
SLC5A11	SGLT	1.30	up	0.01
SLC5A2	SGLT	1.00	up	0.98
SLC5A10	SGLT	1.08	down	0.37

Change information of glucose transporters in acidosis-adapted PANC1 cells.

Supplementary Table 3. Metabolites and genes significantly altered in acidic microenvironment

The metabolites with the most obvious changes	Ion mode	The metabolites with the most obvious changes	Ion mode
Sodium lauryl sulfate	up	Glycyl proline	down
N-Phosphohypotaurocyamine	up	Phosphoenolpyruvate	down
6,7-Dihydro-4-(hydroxymethyl)-2-(p-hydroxyphenethyl)-7-methyl-5H-2-pyrindinium	up	Catechol	down
D-Glucose	up	Glycerol 3-phosphate	down
Resorcinol	up	D-fructose-1,6-bisphosphate	down
Eszopiclone	up	6,7-Dihydro-4-(hydroxymethyl)-2-(p-hydroxyphenethyl)-7-methyl-5H-2-pyrindinium	down
Sulfanilamide	up	Citraconic acid	down
Amifloxacin	up	Ciliatine	down
Butyl 4'-O-butanoyl-6-O-hexadecanoyl-neohesperidoside	up	(Â±)-Glycerol	down
Metanephrine	up	1-monophosphate K salt (1:2)	down
myo-Inositol	up	Saccharopine	down
2-C-Methyl-D-erythritol 4-phosphate	up	D-Glucose	down
Orotic acid	down	2-C-Methyl-D-erythritol 4-phosphate	down
Methanephosphonothioic acid	down	O-phosphoethanolamine	down

Benzylalcohol 2-imidazolidinone	down	Boric acid	down
Amidosulfonic acid	down	Sulfanilamide	down
D-fructose-1-phosphate	down	5-thymidylic acid	down
Ferulic acid Metabolites	down	Amifloxacin	down
3,17,20-trihydroxy-pregn-5-en-11-one	down	Ethanolamine	down
Citric acid	down	Alpha tocopherol	down
3-phosphoglyceric acid	down	6,7-dimethyl-4-hydroxy-2-pteridinamine	down
Aminomethylphosphonic acid	down	Isohexonic acid	down
Glucose-6-phosphate	down	Glycerol	down
Pyrophosphate	down	D-fructose-6-phosphate	down
Formononetin	down	Adenosine-5'-monophosphate	down
Udp-glucuronic acid	down	Dihydroxyacetone phosphate	down
Bisphenol a monomethyl ether	down		
**The 10 most significantly upregulated genes**	**FC (abs)**	**The 10 most significantly downregulated genes**	**FC (abs)**
MMP1	73.51366	CACNA2D1	42.65548
ERVV-2	58.36932	NKAIN4	42.05671
MAGEA2B	46.08178	LONRF2	31.6322
MAGEA6	43.85674	BMP4	30.53697
C1orf21	43.04937	COL5A1	26.27107
MAGEA12	36.03173	INHBB	25.4396
PALMD	29.57542	MB	24.3969
TMEM171	26.02824	PCDHA12	23.88868
TXNIP	24.53838	GFPT2	22.8702
PRSS12	21.07967	FLRT2	21.39978

53 significantly altered metabolites and 20 significantly altered genes in the acidic microenvironment. The greater the difference multiple of FC (ABS) value, the greater the difference between the two samples. The screening criteria are: FC (ABS) is more than 2.0 times, and $P \leq 0.05$.
